# Is there an optimal timing for surgical treatment of pediatric supracondylar humerus fractures in the first 24 hours?

**DOI:** 10.1186/s13018-021-02638-5

**Published:** 2021-08-10

**Authors:** Mustafa Caner Okkaoglu, Fırat Emin Ozdemir, Erdi Ozdemir, Mert Karaduman, Ahmet Ates, Murat Altay

**Affiliations:** Department of Orthopedics and Traumatology, University of Health Sciences, Ankara Kecioren Training and Research Hospital, Pınarbaşı District, Sanatoryum Street, D:25, 06280, Keçiören, Ankara, Turkey

**Keywords:** Surgical timing, supracondylar humerus fracture, working hour

## Abstract

**Background:**

We aimed to determine the ideal surgical timing in the first 24 hours after admission to the hospital of pediatric supracondylar humerus fractures (SHF) that do not require emergent intervention.

**Materials and Methods:**

Patients who underwent surgery in our institution between January 2011 and January 2019 due to pediatric Gartland type 3 SHFs were evaluated retrospectively. Open fractures, fractures associated with vascular injury and compartment syndrome, flexion type fractures were excluded. A total of 150 Gartland type 3 were included. The effect of early (<12 hours) or late (>12 hours) surgical interventions, daytime or night-time surgeries, working or non-working hour surgeries on operative parameters (operative duration and open reduction rate, reduction quality on postoperative early radiographs) were evaluated in pediatric SHFs.

**Results:**

Early (<12 hours) or late (>12 hours), daytime or nighttime, working or non-working hour surgeries were found to be similar in Gartland type 3 patients regarding early postoperative reduction quality, duration of surgery, open reduction rate (p>0.05). Mean times passed from first admission to hospital until surgery were longer in working hour, late (>12 hours) and daytime surgery groups than non-working hour, early (<12 hours) and night-time surgery groups (p<0.001).

**Conclusion:**

Although delaying the operation to the working hours seems to prolong the time until surgery in pediatric Gartland type 3 SHF patients who do not require emergent intervention such as open fractures, neurovascular impairment and compartment syndrome, there may not be a time interval that makes a difference for the patients if surgery is performed within the first 24 hours, thus the surgery could be scheduled according to the surgeons’ preference.

Level of Evidence: Level 3, Retrospective cohort study

## Introduction

Supracondylar humerus fractures (SHF) are the second most common fracture type consisting of about 16% of all pediatric fractures [1]. SHFs are classified according to the direction of the distal segment in the sagittal plane as flexion and extension types. Gartland’s classification is used for categorizing SHFs based on the degree of the displacement: type 1, undisplaced or minimally displaced; type 2, displaced fracture with intact posterior cortical hinge; type 3, completely displaced fracture with an intact periosteal hinge [2].

Surgical treatment of pediatric SHFs aims to reduce the fracture and maintain the reduction quality to preserve the function of the elbow as well as cosmetic appearance [2]. The functional outcomes are mainly dependent on the reduction quality of the fracture [3]. Closed or open reduction and percutaneous pinning is the preferred method of treatment of Gartland type 3 SHFs [4].

Although the methods used in the surgical treatment of pediatric SHFs have been clearly described, there is a controversy in the literature about the timing of the surgery. Delayed surgical intervention can lead to swelling around the elbow which may cause difficulty during closed reduction of the fracture [5]. Operating the pediatric SHF patients within working or non-working hours is still debating in the literature. In recent studies, mal-union rates were reported to be higher in pediatric SHFs operated in the night hour surgeries and mean operative duration was found to be shorter in daytime surgeries [6, 7]. On the other hand, in a recent article, there was no difference in reduction quality, complications and outcomes between pediatric SHF patients operated during the night or the daytime [8]. The aim of this study is to investigate the optimal surgical timing within the first 24 hours of admission in pediatric SHFs that do not require emergent intervention.

## Materials and Methods

Patients who underwent surgery in our institution due to Gartland type 3 SHF between January 2011 – January 2019 were retrospectively reviewed after obtaining local ethics committee approval. Flexion type SHFs and fractures requiring emergent intervention such as open fractures, vascular injuries, compartment syndrome were excluded from the study. A total of 150 Gartland type 3 SHF patients met the inclusion criteria and were included in the study. A written informed consent was obtained from each patient. The study was conducted in accordance with the principles of the Declaration of Helsinki.

**Fig. 1:**
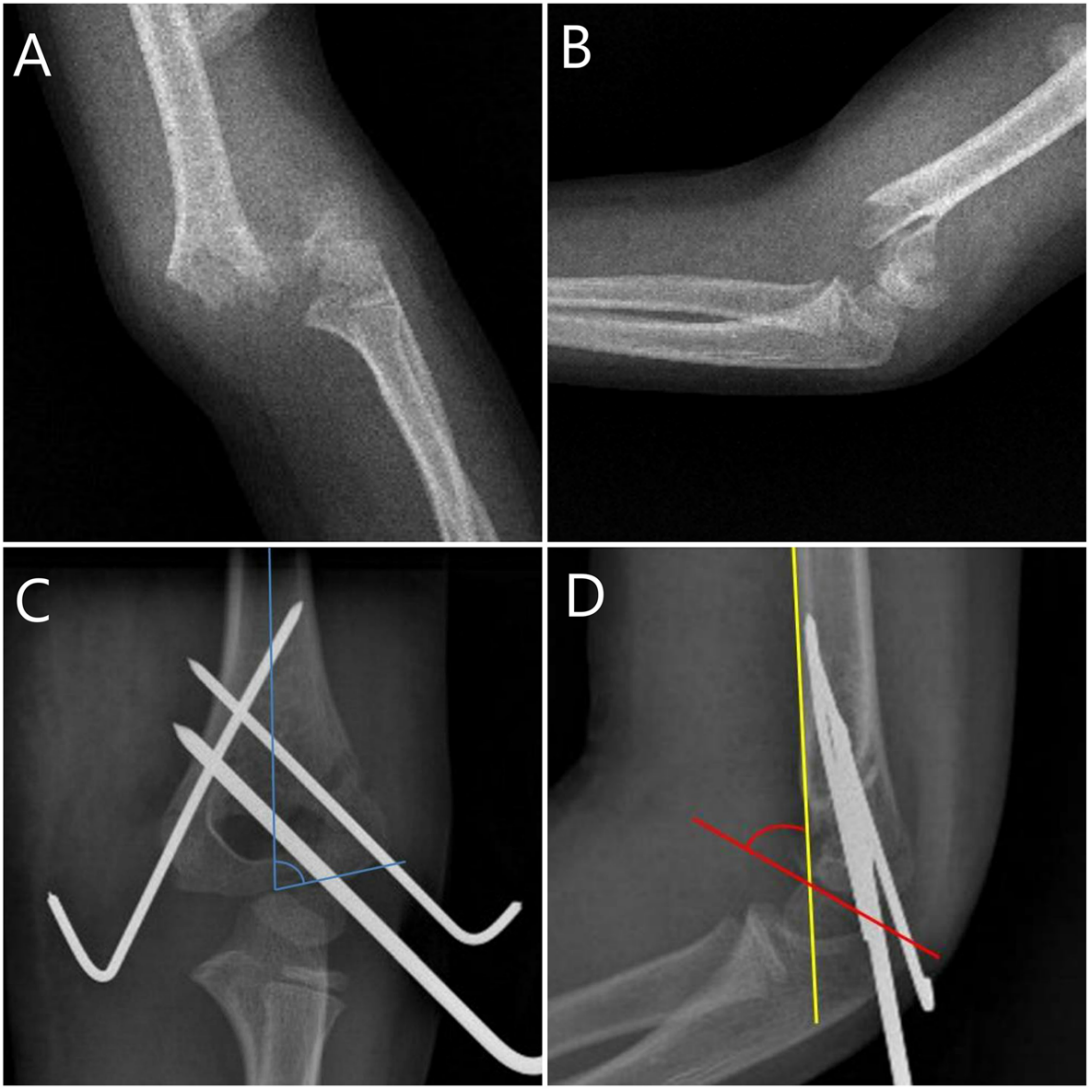
(A) Anteroposterior and (B) lateral elbow radiographs of a 5-year-old female patient with Gartland type 3 supracondylar humerus fracture. (C) The patient was treated with closed reduction and percutaneous pinning. Postoperative Baumann angle was measured by calculating the angle between the longitudinal axis of the humeral shaft and the line along the open capitellar physis. The patient's Bauman angle was calculated as 72 degrees. (D) Postoperative lateral capitellohumeral angle (LCHA) was measured by calculating the angle between the line along the anterior border of the distal humeral shaft (yellow line) and the line along the open capitellar physis (red line) on the lateral radiographs. The patient's LCHA was calculated as 54 degrees. Anterior humeral line (yellow line) passed the mid-third of capitellum (perfect reduction)

Since there is not a separate operating room for trauma cases in our hospital, SHF patients are operated on the night of their admission or the next day in working hours within the first 24 hours. The operations are performed by a senior orthopedic surgeon accompanied by one or two residents. Initially, closed reduction was tried for all the patients under fluoroscopic guidance. Inability to achieve satisfactory closed reduction was the indication for open reduction. Following the reduction of the fracture, percutaneous pinning was performed [9]. It needs to be noted that on non-working hours conditions, less experienced staff in orthopedics work in our operating room. Working hours in our hospital are between 08.00-17.00 on weekdays. For this reason, the patients were divided into two groups as working hours (08.00-17.00) and non-working hours (17.00-08.00). Patients who were admitted at weekends were excluded because they may affect the daytime parameters. Age, gender, time passed from first admission to hospital until surgery, operative duration and open reduction rates were collected from the hospital registry notes. The operative duration was considered as the time between anesthesia given and discontinuation. The reduction quality of the patients was assessed with lateral capitellohumeral angle (LCHA), Baumann angle, anterior humeral line on post-operative early radiographs. The normal range of LCHA was accepted between 22-70 degrees [10] and Baumann angle normal range was accepted as 56-86 degrees [10]. If the anterior humeral line passes the mid-third of capitellum, it was considered as perfect reduction. If the anterior humeral line passes capitellum but outside of mid-third, it was considered as acceptable reduction and those who do not pass the capitellum were considered as poor reduction. (Fig. 1)

Non-working hours were divided into two intervals between 17.00-24.00 (night) and 24.00-08.00 (late night), and compared with the patients operated in working hours on the above-mentioned parameters as a separate cohort. In addition, patients who were operated within first 12 hours (early) or after 12 hours (late) from their admission to surgery were compared with the aforementioned parameters.

The normality analysis of the data was performed with Kolmogorov Smirnov test. The mean values ​​of the normally distributed values ​​were evaluated using the t test, and the mean of the variables that did not show normal distribution was evaluated with Mann Whitney-U test, categorical variables were compared with chi-square test using SPSS 23v. Continuous variables are presented as mean ± SD, whereas categorical variables are given as frequencies. A priori power analysis revealed that utilizing an alpha value of 0.05, beta of 0.80, and a standardized Cohen's d value of 0.5, the estimated sample size required at least 64 patients per cohort or 128 total patients to obtain 0.8 actual power.

## Results

A total of 79 Gartland type 3 SHFs were operated within the working hours while 71 Gartland type 3 SHFs were operated in non-workings. There was no difference in age and sex distribution of the patients who were operated in working or non-working hours (p>0.05). Open reduction rate, mean operation duration and reduction quality at early postoperative radiographs were found to similar between working hour and non-working hour surgeries (p>0.05). However, the mean passed time from first admission until surgery was 14.0 ± 5.1 hours in the working hour surgery group and 6.0 ± 3.5 hours in the non-working hour group (p<0.001). (Table 1)

**Table 1:** Patient demographics and overall results according to working or non-working hours

	Working hours (n=79)	Non-working hours (n=71)	p value
Age	6.4 ± 2.5	5.4 ± 2.8	0.387
Female gender	31/79 (39.2%)	37/71 (52.1%)	0.114
Open reduction	25/79 (31.6%)	20/71 (28.2%)	0.642
Mean operative duration (minutes)	64.7 ± 35.2	58.9 ± 33.0	0.211
Time until surgery (hours)	14.0 ± 5.1	6.0 ± 3.5	<0.001*
Reduction parameters on early postoperative radiographs			
Lateral capitellohumeral angle within normal limits	70/79 (88.6%)	66/71 (93.0%)	0.357
Baumann angle within normal limits	74/79 (93.7%)	67/71 (94.4%)	0.567
Anterior humeral line			0.639
Excellent	18/79 (22.8%)	12/71 (16.9%)	
Acceptable	56/79 (70.9%)	55/71 (77.5%)	
Poor	5/79 (6.3%)	4/71 (5.6%)	

We divided non-working hours into two intervals as 17.00-24.00 (night) and 24.00-08.00 (late night) and we compared the results of these two intervals with daytime surgery (08.00-17.00). There was no statistical difference between daytime surgery and these two subgroups regarding open reduction rate, mean operative duration and reduction qualities at early postoperative radiographs (p>0.05) (Table 2).

**Table 2:** Subgroup analysis according to subintervals of non-working hours

	08.00-17.00 (n=79)	17.00-24.00 (n=51)	p value	08.00-17.00 (n=79)	24.00-08.00 (n=20)	p value
Open reduction	25/79 (31.6%)	12/51 (23.5%)	0.313	25/79 (31.6%)	8/20 (40.0%)	0.484
Mean operative duration (minutes)	64.7 ± 35.2	61.3 ± 36.9	0.394	64.7 ± 35.2	52.7 ± 19.2	0.165
Reduction parameters on early postoperative radiographs						
Lateral capitellohumeral within normal limits	70/79 (88.6%)	47/51 (92.2%)	0.504	70/79 (88.6%)	19/20 (95.0%)	0.357
Baumann angle within normal limits	74/79 (93.7%)	48/51 (94.1%)	0.614	74/79 (93.7%)	19/20 (95.0%)	0.650
Anterior humeral line			0.573			0.932
Excellent	18/79 (22.8%)	8/51 (15.7%)		18/79 (22.8%)	4/20 (20.0%)	
Acceptable Poor	56/79 (70.9%) 5/79 (6.3%)	40/51 (78.4%) 3/51 (5.9%)		56/79 (70.9%) 5/79 (6.3%)	15/20 (75.0%) 1/20 (5.0%)	

We also analyzed the effect of time passed from first admission until surgery on open reduction rate, mean operative duration and reduction quality at early postoperative radiographs. Open reduction rate, mean operative duration and reduction quality of early postoperative radiographs were found to be similar in patients operated within the first 12 hours (early) or after 12 hours (late) (p>0.05) (Table 3).

**Table 3:** Subgroup analysis according to time passed from first admission to hospital until surgery

	<12 hours (n=90)	>12 hours (n=60)	p value
Age	5.8 ± 2.9	6.1 ± 2.5	0.486
Female gender	44/90 (48.9%)	24/60 (40%)	0.284
Open reduction	26/90 (28.9%)	19/60 (31.7%)	0.717
Mean operative duration (minutes)	58.8 ± 30.9	66.7 ± 38.8	0.226
Time until surgery (hours)	6.0 ± 2.9	16.6 ± 2.9	<0.001*
Reduction parameters on early postoperative radiographs			
Lateral capitellohumeral angle within normal limits	82/90 (91.1%)	54/60 (90.0%)	0.819
Baumann angle within normal limits	83/90 (92.2%)	58/60 (96.7%)	0.224
Anterior humeral line			0.867
Excellent	17/90 (18.9%)	13/60 (21.7%)	
Acceptable	68/90 (75.6%)	43/60 (71.6%)	
Poor	5/90 (5.6%)	4/60 (6.6%)	

## Discussion

Surgical timing is one of the recently discussed topics in the treatment of pediatric SHFs. While some authors suggest operating the SHFs as soon as possible, some authors recommend operating these fractures within working hours after providing favorable conditions for both surgeon and non-surgeon factors [5, 11]. In the current study, the optimal surgical timing of Gartland type 3 pediatric SHFs was investigated with early (<12 hours) or late (>12 hours), in daytime or nighttime and on working or non-working hours. None of these timing options were found to create a difference in terms of reduction quality, operative duration, and open reduction rate.

In the literature, there are some studies investigating the optimal surgical timing in other trauma fields besides SHFs and they reported higher complication rates during cases performed at night [12–14]. However, we have reported similar operative parameters on pediatric SHFs operated in the daytime (08.00-17.00) or nighttime (17.00-24.00) and late nighttime (24.00-08.00). As all the surgeries performed by a senior orthopedic surgeon and accompanying one or two residents in our institution regardless of time interval, we believe that performing the surgery as a team may have compensated the less experienced staff’s impact on the surgery at night and late night intervals. This may be the reason that there was no difference on operative parameters at 08.00-17.00 versus 17.00-24.00 and 08.00-17.00 versus 24.00-08.00 intervals.

Controversy exists in the previous literature about the effect of surgical timing of pediatric SHFs on reduction quality. Aydoğmuş et al. showed that patients with SHFs who underwent surgery in non-working hours had poor reduction quality [15]. Paci et al. investigated the results of SHFs operated during working or non-working hours. They reported no difference in terms of operative duration and outcomes, however, mal-union rate was higher in patients operated at night in non-working hours [6]. Yıldırım et al. reported similar reduction quality in pediatric SHFs who were operated in the same or next day of their admission. However, they showed that the likelihood of open reduction increases after 15 hours [16]. We have found no effect of surgical timing on reduction quality. We believe that success at reduction quality is multifactorial and surgical timing is only one of the factors. Different operating room settings, level of experience of operating team and staff, and patient dependent factors such as age, initial swelling of the elbow, obesity and mechanism of the injury may all have an impact on achieving satisfactory reduction. Future studies controlling these confounders are warranted to enlighten the effect of timing on the postoperative reduction quality of SHFs.

Delayed surgeries may lead to higher open reduction rates during surgical treatment of pediatric SHFs. In a systematic review, Loizaou et al. showed that patients had higher open reduction rates who were not operated within the first 12 hours [17]. Walmsley et al. reported higher open reduction rates in Gartland type 3 SHFs operated later than 8 hours (33.3% vs 11.2%) [18]. Sibinski et al. showed no difference between patients who underwent surgery within the first 12 hours and after 12 hours in terms of open reduction rate, operative duration, hospital stay and outcomes [19]. According to the results of the current study, surgical timing was found not affecting the open reduction rates. The controversy regarding the surgical parameters may be due to the varying experience level of operating room staff on orthopedics.

The cut-off point for optimal time passed until surgery has not been clearly defined in the literature. Wenling-Keim et al. reported that the time passed until surgery was not affecting complication rates, but paresthesia was observed more frequently in cases operated between 10 am and 2 pm [20]. In contrast, Abbot et al. demonstrated that the time until surgery does not affect complication rates, operative duration and open reduction rate of pediatric SHFs [11]. Munaghan et al. reported that there was no difference between operating the pediatric SHFs within the first 8 hours or not in terms of operative duration and reduction quality [21]. Prabhakar and Ho showed that there was no difference between the operative duration and fluoroscopy time for those who were operated within the first 15 hours [22]. Kwatkioska et al. reported that there was no clinical and radiological difference between the patients operated within the first 6 hours and those operated after 12 hours [23]. According to the results of the current study, we did not observe any difference in reduction quality, open reduction rate or operative duration in patients who were operated in the first 12 hours or after 12 hours. All the patients were operated within the first 24 hours and this may be the reason for the similar outcomes at different time intervals.

One of the limitations of the current study is its retrospective design, thus randomization of the patients into time intervals was absent. Secondly, all surgical procedures were not performed by the same surgeon. Surgical exposures and pin configurations during fixation were not taken into consideration in the study which may have had an impact on the results. Further controlled studies evaluating the short- and long-term outcomes are needed to define the optimal timing of pediatric SHFs.

## Conclusion

Although delaying the operation to the working hours seems to prolong the time until surgery in pediatric Gartland type 3 SHF patients who do not require emergent intervention such as open fractures, neurovascular impairment and compartment syndrome, there may not be a time interval that makes a difference for the patients if surgery is performed within the first 24 hours, thus the surgery could be scheduled according to the surgeons’ preference.

## Data Availability

The datasets used and/or analysed during the current study are available from the corresponding author on reasonable request

## Abbreviations

**LCHA**: Lateral Capitellohumeral Angle
**SD**: Standard Deviation
**SHF**: Supracondylar Humerus Fracture
